# A survey of study participants’ understanding of informed consent to participate in a randomised controlled trial of acupuncture

**DOI:** 10.1186/s12906-015-0975-y

**Published:** 2016-01-12

**Authors:** Caroline A. Smith, Sarah Fogarty

**Affiliations:** National Institute of Complementary Medicine, Western Sydney University, Locked Bag1797, Penrith, New South Wales 2751 Australia

**Keywords:** Acupuncture, Randomised controlled trial, Ethics, Informed consent

## Abstract

**Background:**

It is important that potential study participants are appropriately informed and understand what is involved with their research participation. A few studies have examined study participants’ understanding of the informed consent process and the adequacy of the information they received when agreeing to participate in a randomised controlled trial. Deficiencies in the consent process have been found. This topic remains an under researched area of acupuncture research. The aim of this study was to examine participants’ understanding of their informed consent and the adequacy of the information presented when agreeing to participate in a randomised controlled trial of acupuncture.

**Methods:**

All women who participated in a randomised controlled trial over an 11 month period were invited to participate in a survey. An anonymous self-completion questionnaire was designed and covered participants’ understanding of informed consent in the clinical trial, their views of the information provided, the opportunity to ask questions, the use of sham acupuncture, their recall of study visits and processes for withdrawal, and their reason for participating in the trial.

**Results:**

A response rate of 59 % was obtained. Over 90 % of subjects indicated there was plenty of opportunity to discuss the study prior to giving consent, and 89 % indicated that questions asked were answered to their satisfaction. The majority of women indicated the amount of information describing acupuncture was about right, however 24 % would have liked more. Information describing sham acupuncture was not considered adequate by 48 % of women, and 35 % would have liked more information, 30 % could not recall why, or were uncertain why a sham group was used. Participants indicated less understanding of the information relating to payment if they became ill due to study participation, risks and discomforts from the study interventions, which of the procedures were experimental and for how long they would be involved in the study.

**Conclusion:**

Trial participants’ understanding of informed consent was overall satisfactory but highlighted some areas of deficiency. Future studies could consider use of supplementary material such as Q and A fact sheets.

## Background

When study participants agree to participate in randomised controlled trials (RCTs) there is no guarantee of a direct benefit to the individual. Therefore it is important that potential participants are fully informed about what is involved with participating in the study and that they understand the potential risks and benefits, and the processes of a RCT. The process of obtaining consent should ensure that the potential participants have been given sufficient time to consider their participation, and the opportunity to discuss the study with others prior to providing informed written consent. International guidelines, regulatory requirements, and the Declaration of Helsinki [1] describe this structure and process as the participant information and consent form. These guidelines detail the requirements of administering informed consent stressing the participants’ understanding of the information and their freedom to decide independently to participate in a study. The following aspects have been identified as essential for informed consent competence disclosure, understanding, voluntariness and consent [2].

Many studies have examined study participants’ understanding of the informed consent process and the adequacy of the information they received when considering participation in a RCT. Deficiencies in the consent process were common. Previous research reported by Brown and colleagues found that half of study participants could not recall facts about randomisation and the processes for withdrawing from the study [3]. An Australian study of oncology patients found 43 % correctly understood the process of randomisation, they thought the allocation to a group was made by chance, and 74 % agreed with the statement *in a clinical trial the doctor would make sure I got the best of the treatments* [4]. An Italian study examining informed consent reported 62 % understood the study procedures and the nature of the trial [5].

The informed consent process has not been well studied in relation to acupuncture studies. Hughes and colleagues examined the consent process in a RCT of sham acupressure which did not explicitly indicate that participants may receive a sham intervention [6]. Participants in this study indicated that they believed they were fully informed when giving consent that it was acceptable to use a sham intervention, and that the majority indicated they assumed one of the treatment arms would be a placebo. In relation to acupuncture this remains an under-researched area, it is important to ensure that the conduct of acupuncture research meets the requirements of ethically sound research.

The ACU-ART RCT is a multi-centre randomised controlled trial currently being implemented in Australia and New Zealand and is examining the role of acupuncture as an adjunct to in vitro fertilisation (IVF) to improve live birth rates [7]. This trial is a parallel RCT of acupuncture compared with a sham control using a non-invasive placebo needle [8]. The placebo needle has a retractable needle shaft, a blunt tip, and skin penetration does not occur, and the needles have a supporting device. The location of sham non-acupuncture points are away from real points and are described in relation to anatomical landmarks and relationship to acupuncture channels. The study is co-ordinated centrally from Western Sydney University Australia, and to date eighteen IVF units have joined the multi-centre trial. We utilised a lead human research ethics committee (HREC) patient information consent form which covered nine recruitment sites, and is comprised of three pages. Separate information and consent forms have been approved by other site specific HREC requirements, and the quantity of information presented varies over three to eight pages. The participant information form describes the rationale for the study, explains to participants that they may be randomised to one of two groups, acupuncture or sham control, the potential risks and benefits including highlighting the risk of side effects from treatment. Research nurses are responsible for providing information to women, and follow up to obtain informed consent. The trial co-ordinator with a background in acupuncture and research methods is available to provide additional information on the study to study participants, with a subsequent conversation by phone or email following randomisation to the trial. This usually provides an opportunity for women to ask and to have answered additional questions about the study or acupuncture if these were not addressed by the research nurse. Study participants, reproductive medicine health care providers, the outcome assessors, and the analyst are blind to study group allocation. The aim of this side study was to examine participants’ understanding of their informed consent and the adequacy of the information presented particularly relating to randomisation and blinding.

## Methods

Women eligible to participate in the clinical trial are aged less than 43 years undergoing a fresh IVF or intracytoplasmic sperm injection (ICSI) cycle, and not currently receiving acupuncture. Participants received an initial study treatment between days 6–8 of their stimulated IVF cycle. All women who were randomised to the ACU-ART trial between December 2013 and November 2014 were invited to participate in this study. An amendment was submitted and approved from the University of Western Sydney Human Ethics Committee (H8936) and nine collaborating IVF unit Human Ethics Committees.

An anonymous self-completion questionnaire was designed and covered different aspects of the informed consent process and information relating to the participant information sheet. We utilised some questions used in previous studies [5, 9–11] despite these instruments not undergoing extensive validity and reliability testing. The questionnaire used a combination of closed and open questions. Closed questions included the subject’s reason for participating in the trial, the opportunity to ask questions, awareness of the risks and side effects, the use of sham acupuncture, their recall of the number of study visits required, the process of withdrawing from the trial, and demographic questions including age, place of residence and education. Questions examined opinions relating to what their participation in the trial involved, and responses to these questions were scored along a five point Likert scale ranging from one- I didn’t understand at all, to five- I understood very well. Open questions examined participants’ understanding and recall of terms such as randomisation and sham acupuncture (Copy of questionnaire see Appendix 1). The questionnaire was sent by post or email from the trial co-ordinator based at the University of Western Sydney. Statistical analyses were carried out using SPSS software (the IBM Statistical Package for the Social Sciences 19, Chicago, IL). Descriptive statistics, using frequencies and percentages were used to describe the data.

## Results

A total of 353 questionnaires were sent out and responses were received from 210 participants giving a response rate of 59 %. Responses to the survey indicated representation across Australian States and Territories, and from our three overseas recruitment sites in New Zealand. The majority of women completing the survey were aged over 35 years, and the majority were highly educated (Table 1).

**Table 1:** Demographic characteristics of study participants

Demographics	Survey population	
	*n* = 210	%
Age (year)		
<35	74	35.2
35–44	120	57.1
Missing data	14	6.6
Place of residence		
New Zealand	69	32.8
South Australia	45	21.4
New South Wales	40	19.0
Victoria	36	17.1
Australian Capital Territory	11	5.2
Queensland	7	3.3
Missing	2	0.9
Education		
Did not finish high school	9	4.2
High School Certificate	22	10.4
Vocational training	42	20.0
University degree	121	57.6
Missing	16	7.6

All women indicated the primary reason for participating in the study was the they hoped acupuncture would improve their chance of having a baby. In addition 61 women (29 %) indicated the importance of undertaking research as a key reason for joining the trial. We sought women’s views about the information they received and the opportunity to ask questions when considering their involvement in the study (Table 2). Over 90 % indicated there was enough opportunity to discuss the study with the clinical staff before deciding to join the trial, and 89 % indicated that any questions they asked were answered to their satisfaction. The majority of women indicated the information describing acupuncture was about right, however 48 (24 %) would have liked more (Table 2). Information describing sham acupuncture was not considered adequate by approximately half of the women, and 35 % would have liked more information. The role of educational status was examined in response these questions and we found women with tertiary education would have liked more information on acupuncture (*p* < 0.009), no other associations were found.

**Table 2:** Views of information presented on the study

Participation in the trial	*N* = 210	%
Opportunity to ask question		
Yes plenty of time	191	91.0
No little opportunity	11	5.2
I can’t remember	8	3.8
Questions answered to your satisfaction		
Yes completely	140	66.7
Yes mainly	49	23.3
No I wasn’t happy with the answers	1	0.5
I didn’t ask any questions	17	8.1
I can’t remember	3	3.8
Information on acupuncture (*n* = 198)		
It was about right	142	71.7
I would have liked more	48	24.2
Information too detailed	4	2.0
I can’t remember	4	2.0
Information on sham acupuncture (*n* = 198)		
It was about right	104	52.5
I would have liked more	70	35.4
I can’t remember	20	10.1
Information too detailed	4	2.0

## Understanding of the patient information

We asked about participants’ understanding of the study based upon the written information they received and follow up verbal interaction with study staff (Table 3 Appendix 1, question 5). The majority of participants clearly understood what the study involved with overall mean scores of 4 in response to these questions. There were five areas in which participants indicated less understanding, these related to payment if they became ill due to their participation in the study, risks and discomforts from the study interventions, which of the procedures were experimental and for how long they would be involved in the study. In addition, over 75 % recalled they were required to attend for treatment on two occasions, and 50 % could accurately recall when they were required to complete questionnaires.

**Table 3:** Participants understanding of the study

*n* = 199	Mean	SD
The fact that participation is voluntary	4.6	1.0
That your treatment involved research	4.5	1.0
What the researchers were trying to find out	4.3	1.0
Treatments and procedures you would undergo	4.3	1.0
How your participation may benefit future patients	4.3	1.0
Possible benefits from participating in the trial	4.2	1.1
Overall how well you understood the study when you signed the consent form	4.2	1.0
Who to contact if you have questions	4.3	1.1
Possible trial risks and discomforts	3.8	1.2
How long you would be in the trial	3.8	1.1
Which treatments were experimental	3.7	1.2
Alternatives to participation in the trial	3.6	1.4
Who will pay for treatment if you become ill/injured	3.0	1.5

We explored participants’ understanding of selected areas relating to questions about the trial and the research terminology used in the patient information sheets through open-ended questions. Participant responses indicated some deficiencies. Over 82 % of responses indicated women could recall what a RCT meant. Responses were illustrated by the following quotations *“I will be selected at random to be in the real or sham acupuncture group to assess the effect of acupuncture on live birth rates”*. Many participants responded indicating their understanding of randomisation and blinding, for example, *“that the person is randomly picked to be in the sham or real acupuncture group”, “I will be allocated to either a treatment or a control group, blind study means I won’t be aware of which group I was allocated to”*. Some subjects responded by discussing the randomisation process, “*its like a name in a hat you get randomly drawn to have either real or placebo acupuncture”,* and *“ where a computer randomly selects which group you will be in”*. Incorrect responses reflected responses relating to blinding *“ no one knows which group you are in”, participants receive either sham or actual treatment without knowing which one”*, or general responses relating to the purpose of the study “*its a experiment used to test the effectiveness of something in this case the acupuncture meridians on the pregnancy success rates in IVF patients”.*

We asked why was sham acupuncture used? Thirty percent could not recall why or were uncertain, and 69 % provided an explanation. Many described it in terms of a non-specific effect relating it to the placebo effect, *“because it was the placebo treatment, you need to see whether the outcomes would be affected by receiving acupuncture or not”, “to rule out the placebo effect, or to rule it in”, “to see if it is all in the mind”*. Others indicated their understanding of the use of a control group, *“ to determine if real acupuncture does actually cause more pregnancies”, “as a control group to eliminate changes caused by bias or belief”, “to see if specific points had results that can be compared against the sham results”. “Sham acupuncture is used to control for the physiological and psychological effects”.*

Fifty nine percent of participants indicated they could not recall the process to withdraw from the study with an additional 16 % responding they were unclear. Of those indicating they understood the process for withdrawing, a small number mentioned they could withdraw any time, however the majority of participants responded to this question by explaining they were of aware of the process to contact the site research nurse or the trial co-ordinator to communicate their intention to withdraw.

Recall of the potential risks and side effects was poor. Over 41 % could not describe any side effects or risks and a further 29 % were unsure. Twenty eight percent of women listed side effects, with the majority accurately recalling acupuncture side effects listed on the information sheet including slight discomfort, pain from needling, and the less frequently occurring events relation to feeling lighted heads, nausea, and dizziness. Five inaccurately recalled a risk of infection.

## Discussion

Our results indicate that trial participants’ understanding of informed consent was overall satisfactory but highlighted some areas where our expectations were not met. Womens’ primary motivation for deciding to join the study was for the potential benefit of improving their chances of success from their IVF cycle although the importance of undertaking the research was additionally highlighted by many women. Overall their understanding of aspects about their trial participation was high, although five areas were highlighted as areas of concern.

Participants felt they had the opportunity to ask questions and generally these were met to women’s satisfaction. Women’s understanding of why sham acupuncture was used was satisfactory however many women would have liked additional information on both acupuncture and sham acupuncture beyond that provided in the participant information form. Further questioning also confirmed a lack of understanding, or recall about potential side effects that could arise, and the processes for withdrawing from the trial. The terms randomisation and blinding appear to be well understood. The variation of women’s response to this question however reflects the individual’s experiences and their responses to this standardised process.

Our findings indicate high levels of research literacy regarding terminology which could have been influenced by the fact that many of our participants had a tertiary education, and perhaps may be more familiar with some of the research terminology frequently used. Responses to those questions indicating a lack of understanding may be attributed to observations that written information is in fact not well read by some participants, or is only partially understood. Information on this trial is generally presented at the start of an IVF cycle, which coincides with a significant amount of complex information being presented to patients in relation to their medical treatment. This may have influenced women’s comprehension and capacity to retain or recall information that was presented to them about the trial. Despite the potential for an overload of information being presented we found there was high satisfaction with women’s questions being well answered by the trial co-ordinator or research nurses. However, our findings indicate there were some areas that could be re-enforced in subsequent conversations or with the provision of additional supplementary material, this is important to ensure that the trial is being conducted in a sound ethical manner.

Our findings concur with some authors and differ to others. Studies have frequently reported that participants have a poor understanding of research side effects, and other trial processes [5, 12, 13]. However contrary to other studies [14, 15] we found that our participants had a good understanding of randomisation. This may be explained by the relatively higher education attributes of our study population. Participants vary in their information needs, and this has been explored and tested in a simple intervention by Kass et al. to improve informed consent [16]. Their study demonstrated that bulleted fact sheets and a Q and A session demonstrated a greater understanding than the standard process of a written patient information form. These communication formats are more desirable rather that adding more information to standard participant information and consent forms which are already long, and can vary in their format and the identification of information that is most important. Nishimura and colleagues in their systematic review identified use of enhanced consent forms, and extended discussion led to improved knowledge outcomes, and that multi-media interventions may prove most useful with long term retention of study information [17].

There are several limitations to the study. The time between the trial participation and questionnaire was up to six months for some study participants and recall bias could have been significant for some of these women. Participants’ self-rated their understanding of the trial processes, there is a possibility of responder bias. The inclusion of additional follow up questions for some key areas however allowed us to objectively confirm the participants’ understanding. Our response rate was 59 %, with 41 % choosing not to participate, there maybe selection bias. It is possible that women responding to this survey were self-selected based upon their interest in this side study, had a greater understanding and recall of the processes involved with the study. Non responders may have lost interest with participating in the study due to a negative pregnancy outcome. This non response bias may result in our findings being an over-estimate of the study outcome participant’s understanding of the informed consent process. The survey was anonymous and we are unable to compare the socio-demographics of responders and non responders to determine whether this influenced study participation . Although we utilised several questions from previous studies [5, 9, 10, 12] not all questions have established validity and reliability. The survey comprised of mostly closed questionnaires with a limited number of open ended questions. This limited the opportunity to examine participants’ understanding of the informed consent process in detail. Future research could consider the use of a qualitative study. We also had variable responses to questions with missing data. Participants may have randomly forgotten to complete a question, or they may have refused to answer, this may influence the reliability of our findings. We propose the sample is demographically representative of women undertaking IVF. We found that the age of women participating in our study is similar to women undergoing IVF in Australia and New Zealand (<35 years 35 % vs 37 %, 35–44 years 57% vs 60 %). We do not have socio-demographic data from non-responders to our survey due to the anonymous nature of the questionnaire, however we found that the higher educational qualifications of women who participated in this side study compared with the trial as a whole were similar (University educated 57 % vs. 57 %, vocational training 20 % vs. 25 %), although there was a greater disparity between those reported not completing high school (4.2 % vs. 9 %). On the basis of these data we propose that the findings reflect the broader population of women undergoing ART.

The language used in the participant information and consent form seems acceptable and we do not have concerns about scientific literacy in relation to the scientific terms used and how they were explained. Our results do highlight an opportunity to consider how to provide additional information in future studies, and that a feasible strategy could take the form of supplementary material such as Q and A sheets that are made available to trial participants at no significant cost to the researchers or the clinical setting. This approach could be explored with human ethics committees and researchers.

## Conclusion

We have no concerns that informed consent has been seriously compromised in this study, or that this study was not ethically sound. Overall our findings suggest the need for written information to go beyond explaining the mechanisms of the trial, and highlight a need for explaining how and why things are done, this can be addressed by having additional Q and A fact sheets.

## Abbreviations

RCT: Randomised controlled trial
IVF: In vitro fertilisation
ICSI: Intracytoplasmic sperm injection
